# LncRNA NORAD Promotes Vascular Endothelial Cell Injury and Atherosclerosis Through Suppressing *VEGF* Gene Transcription *via* Enhancing H3K9 Deacetylation by Recruiting HDAC6

**DOI:** 10.3389/fcell.2021.701628

**Published:** 2021-07-09

**Authors:** Huihua Kai, Qiyong Wu, Ruohan Yin, Xiaoqiang Tang, Haifeng Shi, Tao Wang, Ming Zhang, Changjie Pan

**Affiliations:** ^1^Department of Radiology, Changzhou Second People’s Hospital Affiliated to Nanjing Medical University, Changzhou, China; ^2^Department of Thoracic and Cardiac Surgery, Changzhou Second People’s Hospital Affiliated to Nanjing Medical University, Changzhou, China

**Keywords:** coronary artery disease, atherosclerosis, vascular endothelial cell injury, lncRNA NORAD, VEGF, histone deacetylation

## Abstract

Coronary artery disease (CAD) is a major atherosclerotic cardiovascular disease and the leading cause of mortality globally. Long non-coding RNAs (lncRNAs) play crucial roles in CAD development. To date, the effect of lncRNA non-coding RNA activated by DNA damage (NORAD) on atherosclerosis in CAD remains unclear. The primary aim of this study was to investigate the effect of lncRNA NORAD on vascular endothelial cell injury and atherosclerosis. Here, ox-LDL-treated human umbilical vein endothelial cells (HUVECs) and high-fat-diet (HFD)-fed ApoE^–/–^ mice were utilized as *in vitro* and *in vivo* models. The present study found that lncRNA NORAD expression was increased in ox-LDL-treated HUVECs and thoracic aorta of atherosclerotic mice, and knockdown of lncRNA NORAD alleviated vascular endothelial cell injury and atherosclerosis development *in vitro* and *in vivo*. Knockdown of lncRNA NORAD aggravated ox-LDL-reduced or atherosclerosis-decreased vascular endothelial growth factor (VEGF) expression in HUVECs and thoracic aorta of mice to ameliorate vascular endothelial cell injury and atherosclerosis development. Moreover, nucleus lncRNA NORAD suppressed *VEGF* gene transcription through enhancing H3K9 deacetylation *via* recruiting HDAC6 to the *VEGF* gene promoter in ox-LDL-treated HUVECs. In addition, VEGF reduced FUS (FUS RNA binding protein) expression by a negative feedback regulation in HUVECs. In summary, lncRNA NORAD enhanced vascular endothelial cell injury and atherosclerosis through suppressing *VEGF* gene transcription *via* enhancing H3K9 deacetylation by recruiting HDAC6. The findings could facilitate discovering novel diagnostic markers and therapeutic targets for CAD.

## Introduction

Coronary artery disease (CAD) is a major cardiovascular disease, which is considered as the leading cause of mortality globally (Khera and Kathiresan, 2017; Malakar et al., 2019). According to a Chinese Ministry of Health report, more than 1 million people die from CAD in China annually (Wang et al., 2017). In United States, about 6 million deaths occur due to CAD each year (Lloyd-Jones et al., 2009). What is worse, the incidence of CAD rises rapidly (Lloyd-Jones et al., 2009). Therefore, CAD has been a major global health concern. It is urgent to discover novel diagnostic markers and therapeutic targets for CAD.

CAD is an inflammation atherosclerotic disease (Ross, 1999). It has been proved that endothelial cell injury or endothelial dysfunction is the initial step of the atherosclerosis process (Matsuzawa and Lerman, 2014; Jensen and Mehta, 2016; Qin et al., 2018). Growing evidence have indicated that long non-coding RNAs (lncRNAs) play crucial roles in endothelial cell injury, atherosclerosis, and CAD. Fox example, knockdown of lncRNA TTTY15 attenuates vascular endothelial cell injury in cardiovascular disease through targeting miR-186-5p (Zheng et al., 2020). Moreover, lncRNA nexilin F-actin binding protein antisense RNA 1 (NEXN-AS1) ameliorates atherosclerosis through promoting NEXN expression (Hu et al., 2019). In addition, lncRNA ANRIL might mitigate CAD regulating NF-κB pathway *via* miR-181b (Guo et al., 2018). However, the knowledge of the role of lncRNAs in endothelial cell injury, atherosclerosis, and CAD is still limited.

LncRNA non-coding RNA activated by DNA damage (NORAD) is a newly identified lncRNA. Previous studies have indicated that lncRNA NORAD is involved in maintenance of genomic stability and regulation of mitosis. For instance, lncRNA NORAD binds with RBMX in nucleus to assemble a ribonucleoprotein complex, which is termed NORAD-activated ribonucleoprotein complex 1 (NARC1), and maintains the genome stability through NARC1 (Munschauer et al., 2018). In addition, cytoplasmic lncRNA NORAD interacts with PUMILIO protein to modulate cell mitosis through regulating levels of PUMILIO-targeted mRNAs (Tichon et al., 2016). Besides, lncRNA NORAD contributes to the progression of multiple tumors. A recent study has revealed that Yap-repressed lncRNA NORAD inhibits lung and breast tumor metastasis through binding and sequestering S100P (Tan et al., 2019). Moreover, lncRNA NORAD enhances pancreatic cancer metastasis through promoting epithelial–mesenchymal transition *via* targeting hsa-miR-125a-3p to increase RhoA expression (Li et al., 2017). In addition, a few recent studies have revealed that lncRNA NORAD contributes to vascular endothelial cell injury and atherosclerosis (Bian et al., 2020; Zhao et al., 2020). However, the mechanisms on how lncRNA NORAD regulates endothelial cell injury, atherosclerosis, and CAD remain unclear.

Vascular endothelial growth factor (VEGF) is a recognized growth factor playing pivotal roles in angiogenesis through increasing the proliferation rate and activities of endothelial progenitor cells (EPCs) (Yancopoulos et al., 2000; Jin et al., 2015). Angiogenesis could effectively relieve atherosclerotic diseases including CAD (Camare et al., 2017; Wu et al., 2018; Ma Q. et al., 2020), while endothelial cell injury and endothelial dysfunction impair angiogenesis (Xing et al., 2017; Ungvari et al., 2018). Numerous studies have demonstrated that VEGF could attenuate endothelial cell injury or endothelial dysfunction to relieve the pathogenesis of patients with CAD (Jin et al., 2015; Longchamp et al., 2018; Liang et al., 2020). In addition, VEGF mRNA level is decreased in CAD patients (Amoli et al., 2012), and serum VEGF level is a potential indicator of the severity of CAD (Kucukardali et al., 2008). However, no studies have reported the role of lncRNA NORAD in VEGF expression and function in endothelial cell injury, atherosclerosis, and CAD.

It was hypothesized that lncRNA NORAD may regulate vascular endothelial cell injury and atherosclerosis through VEGF. Thus, the primary aim of this study was to investigate the effects of lncRNA NORAD on vascular endothelial cell injury and atherosclerosis and potential mechanisms.

## Materials and Methods

### Serum Collection

All experimental procedures in human were approved by the Ethic Committee of Changzhou Second People’s Hospital Affiliated to Nanjing Medical University. Written informed consent was obtained from all participants enrolled in this study. Fasting venous blood was drawn from 15 healthy controls and 15 CAD patients recruited for this study and centrifuged to collect serum; subsequently, the separated serum was stored at –80°C until detection.

### Cell Culture

Human umbilical vein endothelial cells (HUVECs) were obtained from the Cell Bank at the Chinese Academy of Sciences (Shanghai, China). Cells were cultured in DMEM-high glucose medium (Hyclone, United States) containing penicillin (100 units/ml, Hyclone, United States) and fetal bovine serum (10%, Hyclone, United States), at 37°C and 5% CO_2_.

### Lentivirus Packaging

Short hairpin RNA (shRNA) sequence targeting lncRNA NORAD and a control shRNA were designed according to principles (Table 1). Next, two single DNA strands of shRNAs were synthesized by Hechuang Biotechnology Co., Ltd. and then ligated with lentivirus interference vector pLVX-shRNA2 to construct lentivirus shRNA interference vector of lncRNA NORAD (shNORAD) and control lentivirus shRNA vector (siCtrl). Subsequently, shNORAD or siCtrl was co-transfected with lentivirus packaging helper plasmid lenti-X HTx into 293 FT cells by Lipofectamine 2000 (Invitrogen, United States) for lentivirus packaging. Finally, the lentivirus stock solution was collected and concentrated and the virus titer was determined.

**TABLE 1:** shRNA sequence.

	Sense (5′-3′)	Antisense (5′-3′)
shNORAD	GAUGUAUGUUAAAUGUAUA	UAUACAUUUAACAUACAUC
siCtrl	TUUCUCCGAACGUGUCACGU TTTTCAAGAGA	ACGUGACACGUUCGGAGAA TTTTTTTT

### Cell Treatment

To mimic vascular endothelial cell injury in atherosclerosis, HUVECs were treated with 100 μg/ml ox-LDL (Yeasen, Shanghai, China) for 24 h (Li et al., 2019; Su et al., 2021). For RNA interference of HDAC6 and FUS, small interfering RNA (siRNA) targeting HDAC6 (siHDAC6) or FUS (siFUS) and negative control (siCtrl) were synthesized by Hechuang Biotechnology Co., Ltd., and transfected into HUVECs using Lipofectamine 2000. For lentivirus interference, HUVECs were infected with shNORAD or siCtrl.

### ELISA

The levels of von Willebrand factor (vWF), stumpy (sTM), sE-selectin, and solution vascular cell adhesion molecule 1 (sVCAM-1) in cell medium or serum of mice were detected by vWF ELISA kit, sTM ELISA kit, sE-selectin ELISA kit, and sVCAM-1 ELISA kit purchased from Beijing Wintersong Biotech (Beijing, China) according to the manufacturer’s instruction. Briefly, washing buffer was diluted with deionized water to 1× application buffer. Next, 50-μl standards with different concentrations and 10-μl samples were added into wells of a 96-well plate, respectively. Triplicates were made for each sample. Subsequently, 100 μl of enzyme-labeled reagent was added into each well except for the blank wells, and the plate was incubated at 37°C for 1 h followed by an additional washing (30 s, five times). Then, 50 μl of color reagent A and B were added into each well followed by an incubation at 37°C for 15 min in the dark, and the reaction was terminated by 50 μl of termination solution. Finally, the absorbance (OD value) was measured at 450 nm wavelength.

### Quantitative Reverse Transcription-PCR (qRT-PCR)

Total RNAs (2 μg) from human serum, HUVECs, or mouse thoracic aorta tissues were extracted using TRIZOL (Invitrogen, United States). The first-strand cDNA was made according to the manufacturer’s instructions (TaKaRa Biotechnology, China). The amount of target RNA was normalized to the amount of internal control (GAPDH) and the results were given by 2^–ΔΔ*Ct*^ relative to the control sample. The qRT-PCR was performed by SYBR Green (Takara Biotechnology, China). The primer sequence was as follows: NORAD forward: 5′-CATTGGGCG AGACCTACCTA-3′, reverse: 5′-ACGTGGCCTGTCATTCTA CC-3′; VEGF forward: 5′-GCCCTGAGCCAGGAAATAAA-3′, reverse: 5′-TGGTGGAGGTGCTAGGTTA-3′; GAPDH forward: 5′-AACGGATTTGGTCGTATTGGG-3′, reverse: 5′-CCTGGA AGATGGTGATGGGAT-3′.

### Western Blot

Total proteins were extracted from HUVECs and mouse thoracic aorta tissue using RIPA buffer (CST, United States) and quantified using the BCA Protein Quantification Kit (Abbkine, United States). The same concentration of protein was loaded and separated by SDS-polyacrylamide gel electrophoresis (SDS-PAGE) and then transferred to PVDF membrane (Millipore, United States). Subsequently, membranes were blocked with 5% non-fat milk for 1 h at room temperature (RT) and then incubated with primary antibody at 4°C overnight. After the incubation with primary antibodies, the membranes were washed by Tris-buffered saline containing 0.1% Tween20 (TBST) and then incubated with corresponding second antibodies (1:5,000, BOSTER, China) at RT for 1 h. Finally, the signals of targeted proteins were detected by chemiluminescence detection kit (Beyotime, China). The primary antibodies used in this study included VEGF (1:200, Bioss, China), HDAC6 (1:200, Bioss), FUS (1:200, Bioss), cleaved Caspase 3 (1:500, Abcam, United States), and GAPDH (1:3,000, KangCheng, China).

### Luciferase Reporter Gene System

Firstly, *VEGF* gene promoter/internal enhancer/luciferase reporter gene plasmid was constructed. Next, HUVECs were co-transfected with the reporter gene plasmid and siHDAC6, siFUS, and siCtrl, or transfected with the reporter gene plasmid and infected with shNORAD and shCtrl. After transfection of infection for 48 h, cells were collected and the relative luciferase activity was detected using the dual-luciferase reporter assay system (Promega, United States).

### Chromatin Isolation by RNA Purification (ChIRP)

The antisense probes for the NORAD-specific sequence of lncRNA were synthesized, and then the cells were cross-linked with 1% paraformaldehyde to extract the nuclei. After sonication, the probes and cell lysates were incubated at 4°C overnight and then the lncRNA was captured by biotin affinity magnetic beads. NORAD probe was used to wash out the non-specific binding substance and elute the magnetic beads to obtain the DNA fragment bound to NORAD of lncRNA. Then, qPCR was used to detect the presence of VEGF promoter fragment. The probes used for ChIRP are listed in Table 2.

**TABLE 2:** Probes used for ChIRP.

Gene	Group	No.	Probe sequence
NORAD	ChIRP	1	ACTAATTTGTCCGTTATATATACAA
		2	GAGATGGTCAAACAAATTCCTATGC
		3	TCCGACAGCAAAGTCTGGTAGAATG
		4	GACAGCAAAGTCTGGTAGAATGAAG
Lac Z	NC	1	ACCGCATATGGTGCACTCTC
		2	TCCTTACGCATCTGTGCGGT
		3	GCGAATGGCGCCTGATGCGG
		4	CTTCCCAACAGTTGCGCAGC
		5	ATAGCGAAGAGGCCCGCACC
		6	CACATCCCCCTTTCGCCAGC
		7	GGCGTTACCCAACTTAATCGC
		8	TTACAACGTCGTGACTGGGA
		9	CATGCAAGCTTGGCACTGGC
		10	CCATGATTACGAATTCGAGCTC

### RNA Antisense Purification (RAP)

RAP was performed by the RAP Kit (Hechuang Biotechnology Co., Ltd.). Briefly, HUVECs were cross-linked by 1% formaldehyde for 10 min at RT and then stopped by glycine. Next, HUVECs were lysed by pre-chilled lysis buffer containing protease inhibitor and RNase inhibitor. Shear DNA was removed using DNase salt stock with DNase for 10 min at 37°C. Subsequently, probes and streptavidin magnetic beads were mixed in 0.5 ml of 1× hybridization buffer for 30 min at RT. Then, cell lysis without DNA was added into 1× hybridization buffer and incubated with probes at 45°C for 90–180 min. Following the incubation with probes, streptavidin magnetic beads were collected using a magnetic separation device (Thermo Fisher Scientific), and precipitated complexes were cleaned five times by washing buffer at 35°C. Next, immunoprecipitated proteins were eluted with elution buffer and analyzed by WB using HDAC6 antibody (1:300, Proteintech).

### Co-immunoprecipitation (Co-IP)

HUVECs were lysed by the non-denaturing lysis buffer containing protease inhibitor (Sigma, United States). Next, the supernatant of cell lysis was pre-cleaned with protein A/G magnetic beads (Thermo Fisher Scientific, United States) for 2 h at 4°C. Subsequently, about 300 μg of protein was incubated with 1 μg of FUS antibody (Proteintech, United States) and 25 μl of protein A/G magnetic beads for immunoprecipitation at 4°C overnight. Following the incubation with FUS antibody and protein A/G magnetic beads, protein A/G magnetic beads were collected using a magnetic separation device (Thermo Fisher Scientific), and precipitated complexes were cleansed by washing buffer (Thermo Fisher Scientific). Finally, bound proteins were analyzed by WB using HDAC6 antibody (1:300, Proteintech). Rabbit IgG was used for negative control.

### Chromatin Immunoprecipitation (ChIP)

ChIP was assessed by the ChIP Kit obtained from Hechuang Biotechnology Co., Ltd. In brief, HUVECs were cross-linked by 1% formaldehyde for 15 min at RT and then stopped by glycine. Next, HUVECs were lysed by sonication to shear DNA. Insoluble material was removed from cell lysis by centrifugation. DNA chromatin sample (25 mg) was adjusted to a total volume of 500 ml in 450 ml of the dilution buffer with protease inhibitors. Chromatin samples were then incubated with 1 μg of HDAC antibody (Proteintech), H3K9ac antibody (Active Motif), or anti-rabbit IgG antibody (Cell Signaling Technologies) and incubated with protein A/G magnetic beads overnight at 4°C with gentle rotation. Magnetic beads were collected by magnetic separation device (Thermo Fisher Scientific) and cleaned using washing buffer. Subsequently, immunoprecipitated DNAs were eluted with 100 μl of elution buffer containing Proteinase K at 62°C for 2 h. Then, DNAs were purified utilizing the spin columns and dissolved in the elution buffer. Finally, chromatin DNAs were analyzed by PCR and qPCR. Primers used for ChIP PCR and qPCR are listed in Table 3.

**TABLE 3:** Primers used for ChIP PCR and qPCR.

		Primer sequence	bp
VEGF site 1	F	GGCAAGATCTGGGTGGATAAT	192
	R	CAACTCTCCACATCTTCCCTAAG	
VEGF site 2	F	CCAGACTCCACAGTGCATAC	207
	R	TGGCCTGCAGACATCAAA	
VEGF site 3	F	GGTCACTCCAGGATTCCAATAG	194
	R	GAAACTCTGTCCAGAGACACG	
GAPDH	F	AAAAGCGGGGAGAAAGTAGG	212
	R	AAGAAGATGCGGCTGACTGT	

### Animals and Treatment

Six-week-old male ApoE^–/–^ mice and wild-type (WT) male C57BL/6J mice were purchased from GemPharmatech Co., Ltd. (Nanjing, China). The mice were exposed to standard light conditions at 25°C. ApoE^–/–^ mice were given HFD (21% fat, 0.25% cholesterol) purchased from GemPharmatech Co., Ltd. at the age of 8 weeks. After the ApoE^–/–^ mice were fed with HFD for 8 weeks, they were given lentivirus shNORAD or control shCtrl at a dosage of 2 × 10^9^ pfu/mouse *via* tail vein injection and fed on HFD for another 8 weeks until they were 24 weeks old. The C57BL/6J mice fed with a normal diet were labeled as the WT control group. All animal experiments were performed in accordance with the Ethic Committee of Changzhou Second People’s Hospital Affiliated to Nanjing Medical University.

### Oil Red O Staining

Oil Red O staining was used to assess lipid accumulation in an atherosclerotic plaque. Mice were anesthetized with pentobarbital and euthanized using CO_2_. Subsequently, the whole thoracic aorta was isolated, cut lengthwise with scissors, placed in an Oil Red O solution for 15 min, and immersed in 70% ethanol until the normal tissues became white. The aorta was photographed by an Eclipse ci-l camera microscope (Nikon, Japan), and Oil Red O-positive areas were analyzed using ImageJ.

### Hematoxylin and Eosin (H&E) Staining

Mice were anesthetized with pentobarbital and euthanized using CO_2_. Next, the aortic roots were excised and fixed overnight with 4% paraformaldehyde and then embedded in paraffin wax. The tissues were cut into 5-μm thickness. H&E staining was performed according to the instructions provided by the manufacturer (Solarbio Biotechnology, Beijing, China). The microscopic images of lesions in the aortic sinus were captured by an Eclipse ci-l camera microscope (Nikon). The percentage of lesion area was analyzed using ImageJ.

### Ultrasound Imaging

All mice were fed for more than 8 h 1 day before the test. After injecting anesthetic, mouse abdominal hair was removed by depilatory cream and then mice were cleaned with water. Subsequently, the blood flow velocity, cardiac function analysis, and vessel diameter were measured by a Doppler ultrasound diagnostic instrument (Visualsonics, Toronto, Canada).

### Statistical Analysis

The data were expressed as mean ± SD and analyzed by SPSS 20 software. The unpaired Student’s *t*-test was used to compare two groups while one-way ANOVA was used for statistics of more than three groups. *p* < 0.05 was considered as statistically significant.

## Results

### LncRNA NORAD Is Upregulated in the Serum of CAD Patients and HUVECs Treated With ox-LDL

**FIGURE 1:**
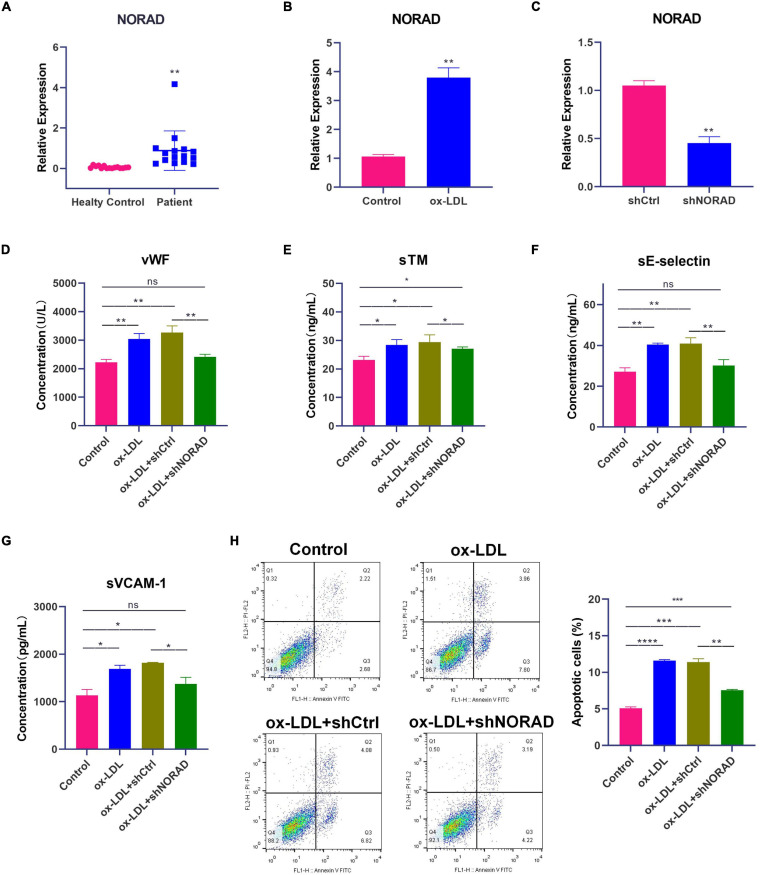
Knockdown of lncRNA NORAD attenuates ox-LDL-induced vascular endothelial injury. **(A)** The level of lncRNA NORAD in serum of healthy controls (*N* = 15) or CAD patients (*N* = 15). **(B)** The level of lncRNA NORAD in HUVECs treated with or without ox-LDL. **(C)** The level of lncRNA NORAD in HUVECs transfected with control shRNA or shRNA-targeting lncRNA NORAD. **(D–G)** The level of vWF **(D)**, sTM **(E)**, sE-selectin **(F)**, and sVCAM-1 **(G)** in HUVECs detected by ELISA. **(H)** Representative images of flow cytometric apoptosis assay in HUVECs. The bar graph showed the quantification of apoptotic cell number in each group. shCtrl, control shRNA; shNORAD, shRNA-targeting lncRNA NORAD. *N* = 3 except in panel **(A)**. *p < 0.05, **p < 0.01, ***p < 0.001, ****p < 0.0001.

First, the level of lncRNA NORAD in the serum of patients with CAD was identified by qPCR. Results indicated that the level of lncRNA NORAD in the serum of CAD patients was significantly increased compared with that in the serum of healthy controls (Figure 1A) (*p* < 0.01). In addition, ox-LDL was utilized to mimic endothelial injury in atherosclerosis. Results showed that lncRNA NORAD expression was increased in ox-LDL-treated HUVECs compared to that in control HUVECs treated without ox-LDL (Figure 1B) (*p* < 0.01). This result was consistent with that of a previous study (Zhao et al., 2020). The above data suggested that endothelial injury, atherosclerosis, and CAD should increase lncRNA NORAD expression.

### Knockdown of LncRNA NORAD Attenuates ox-LDL-Induced Vascular Endothelial Injury

Short hairpin RNA (shRNA)-targeting lncRNA NORAD (shNORAD) was used to knock down the lncRNA NORAD expression in HUVECs. The efficiency of knockdown was verified by qPCR. The lncRNA NORAD expression in HUVECs infected with shNORAD was knocked down by more than 60% compared with that in HUVECs infected with control shRNA (shCtrl) (Figure 1C) (*p* < 0.01). Subsequently, the levels of vascular endothelial injury markers including vWF, sTM, sE-selectin, and sVCAM-1 were detected by ELISA. Results indicated that ox-LDL significantly increased the levels of vWF (*p* < 0.01), sTM (*p* < 0.05), sE-selectin (*p* < 0.01), and sVCAM-1 (*p* < 0.05), while knockdown of lncRNA NORAD dramatically reduced ox-LDL-induced vWF, sTM, sE-selectin, and sVCAM-1 levels (Figures 1D–G). Compared to shNORAD transfection, levels of vWF (*p* < 0.01), sTM (*p* < 0.05), sE-selectin (*p* < 0.01), and sVCAM-1 (*p* < 0.05) were not affected by shCtrl transfection (Figures 1D,E).

Besides, apoptosis of HUVECs was identified by flow cytometric apoptosis assay. Results showed that ox-LDL induced apoptosis of HUVECs (*p* < 0.0001), and lncRNA NORAD knockdown reversed the effect of ox-LDL on apoptosis of HUVECs (Figure 1H). Compared to shNORAD transfection, cell apoptosis was not affected by shCtrl transfection (Figure 1H) (*p* < 0.01). Therefore, these results suggested that ox-LDL induced vascular endothelial injury through upregulating lncRNA NORAD expression.

### Knockdown of LncRNA NORAD Aggravates ox-LDL-Reduced VEGF Expression in HUVECs

**FIGURE 2:**
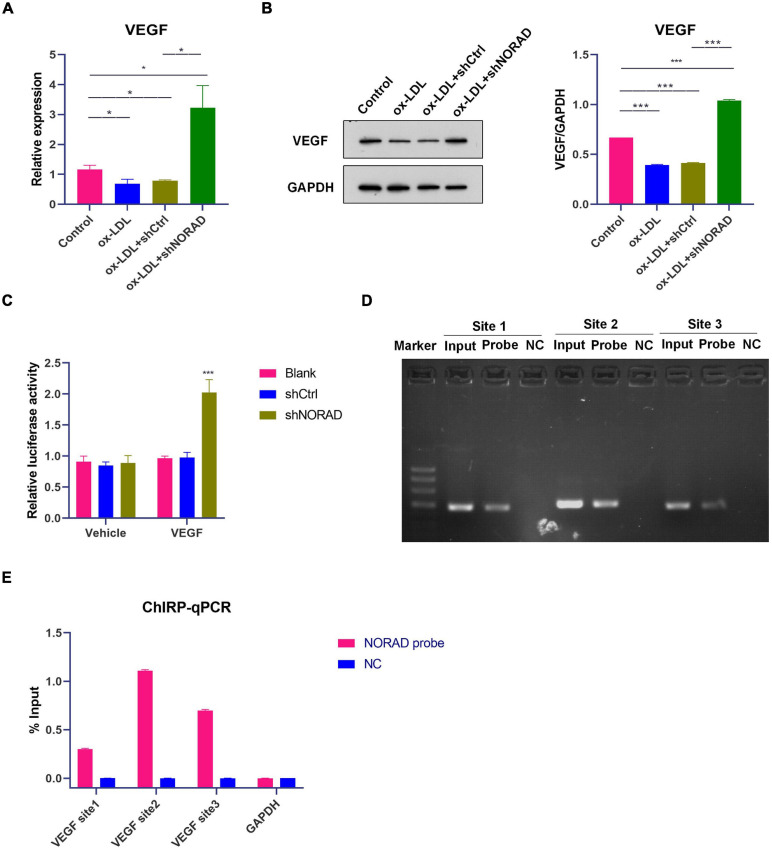
Knockdown of lncRNA NORAD aggravates ox-LDL-reduced VEGF expression in HUVECs. **(A)** The mRNA level of VEGF in HUVECs (*p < 0.05). **(B)** The protein level of VEGF in HUVECs (***p < 0.001). **(C)** *VEGF* gene promoter activity analyzed by relative luciferase reporter activities in HUVECs transfected with control shRNA or shRNA-targeting lncRNA NORAD (***p < 0.001 vs. blank group). **(D)** Immunoprecipitated chromatin associated with lncRNA NORAD was analyzed using ChIRP followed by PCR for the *VEGF* gene promoter in HUVECs. **(E)** Quantification of lncRNA NORAD occupancy on the *VEGF* gene promoter by qRT-PCR in HUVECs. shRNA; shNORAD, shRNA-targeting lncRNA NORAD; NC, negative control. *N* = 3.

To identify the effect of lncRNA NORAD on VEGF expression, qPCR and WB were performed. Results indicated that ox-LDL reduced VEGF mRNA (*p* < 0.05) and protein levels (*p* < 0.001), while knockdown of lncRNA NORAD induced a significant increase in VEGF mRNA and protein levels (Figures 2A,B). Compared to shNORAD transfection, the VEGF mRNA (*p* < 0.05) and protein expression (*p* < 0.001) were not affected by shCtrl transfection (Figures 2A,B). Moreover, luciferase reporter gene assay was performed with transfection of plasmids containing VEGF gene promoter to further identify the role of lncRNA NORAD in VEGF expression. The luciferase activity of cells transfected with plasmids containing *VEGF* gene promoter was increased by the infection of shNORAD compared to that of cells transfected with plasmids containing *VEGF* gene promoter and infected with shCtrl (Figure 2C) (*p* < 0.001). These results suggested that lncRNA NORAD suppressed the transcription of *VEGF* gene.

It has been reported that lncRNA could regulate the transcription of target gene through binding with a target gene promoter (Hu et al., 2018). Thus, ChIRP followed by PCR and qPCR was performed whether lncRNA NORAD interacted with the *VEGF* gene promoter. Primers used for the amplification of three sites in the *VEGF* gene promoter were designed and synthesized. Results showed that lncRNA NORAD occupied all three sites of the *VEGF* gene promoter and most enriched in the second and third sites in ox-LDL-treated HUVECs (Figures 2D,E), suggesting that these two sites should be occupancy sites of lncRNA NORAD at the *VEGF* gene promoter. All these data revealed that lncRNA NORAD suppressed the transcription of the *VEGF* gene *via* interacting with the *VEGF* gene promoter.

### Nucleus LncRNA NORAD Associates With HDAC6 *via* FUS in ox-LDL-Treated HUVECs

**FIGURE 3:**
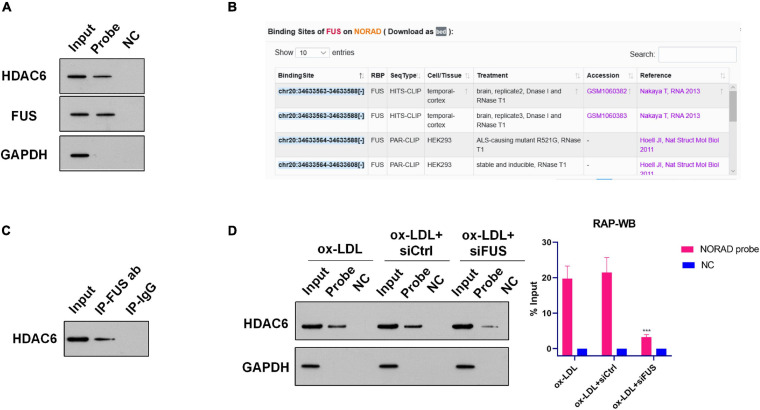
Nucleus lncRNA NORAD associates with HDAC6 *via* FUS in ox-LDL-treated HUVECs. **(A)** Representative images of WB following RAP performed by probe-targeting lncRNA NORAD in HUVECs. **(B)** Potential binding sites for FUS in lncRNA NORAD analyzed by ENCORI. **(C)** Representative images of Co-IP using an anti-FUS antibody in HUVECs. Rabbit IgG was used as negative control. **(D)** Representative images of WB following RAP performed by probe-targeting lncRNA NORAD in HUVECs transfected with control shRNA or shRNA-targeting lncRNA NORAD under ox-LDL treatment. In the bar graph, the protein level of HDAC6 was quantified and normalized by input. shRNA; shNORAD, shRNA-targeting lncRNA NORAD; NC, negative control; siCtrl, control siRNA; siFUS, FUS siRNA (***p < 0.001 vs. other groups). *N* = 3.

Numerous studies have indicated that nucleus lncRNAs regulate the transcription of target genes through associating with histone modification enzymes to alter histone modifications, such as methylation and acetylation (Chu et al., 2012; Jiang et al., 2020; Ma H. et al., 2020). Thus, RAP was performed using the lncRNA NORAD probe to discover histone modification enzymes binding with nucleus lncRNA NORAD in ox-LDL-treated HUVECs. The enrichment of potential histone modification enzymes was assessed by WB. Results of RAP showed that the histone deacetylase 6 (HDAC6) was enriched by the lncRNA NORAD probe (Figure 3A and Supplementary Figure 1A), indicating that HDAC6 associated with nucleus lncRNA NORAD in HUVECs. As a negative control, Laz probe could not enrich HDAC6 (Figure 3A and Supplementary Figure 1A).

However, HDAC6 is not an RNA binding protein (RBP). Therefore, nucleus lncRNA NORAD should interact with HDAC6 *via* other RBPs. Bioinformatics analysis by ENCORI^1^ found that lncRNA NORAD binds with RBP FUS in cancer cells (Figure 3B). Next, RAP was performed to identify whether nucleus lncRNA NORAD interacted with FUS in ox-LDL-treated HUVECs. Results of RAP showed that FUS was enriched by the lncRNA NORAD probe (and Supplementary Figure 1A), which suggested that lncRNA NORAD associated with FUS in ox-LDL-treated HUVECs. Also, Laz probe (NC) could not enrich FUS (Figure 3A and Supplementary Figure 1A).

A previous study has revealed that HDAC6 interacted with FUS in prostate cancer cells (Watson et al., 2016). Here, Co-IP using FUS antibody was performed to determine whether HDAC6 associated with FUS in the nucleus of ox-LDL-treated HUVECs. Results found that HDAC6 was enriched by FUS antibody whereas IgG could not enrich HDAC6 in the nucleus of ox-LDL-treated HUVECs (Figure 3C and Supplementary Figure 2). This result suggested that HDAC6 associated with FUS in the nucleus of ox-LDL-treated HUVECs.

Next, RAP was performed using the lncRNA NORAD probe to identify whether nucleus lncRNA NORAD interacted with HDAC6 *via* FUS. First, FUS was silenced by siRNA (siFUS) (Supplementary Figure 3). Besides, the result of RAP showed that the enrichment of HDAC6 by the lncRNA NORAD probe was dramatically reduced in ox-LDL-treated HUVECs infected with shNORAD compared to those in ox-LDL-treated HUVECs and ox-LDL-treated HUVECs infected with control siRNA (siCtrl) (Figure 3D and Supplementary Figures 1A–C) (*p* < 0.001). All these results together suggested that nucleus lncRNA NORAD associated with HDAC6 *via* FUS in ox-LDL-treated HUVECs.

### Knockdown of HDAC6 or FUS Enhances *VEGF* Gene Transcription in ox-LDL-Treated HUVECs

**FIGURE 4:**
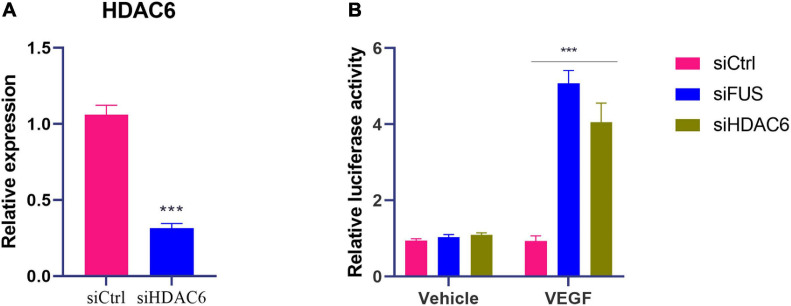
Knockdown of HDAC6 or FUS enhances *VEGF* gene transcription in ox-LDL-treated HUVECs. **(A)** The mRNA level of HDAC6 in HUVECs transfected with control siRNA or HDAC6 siRNA. **(B)** *VEGF* gene promoter activity analyzed by relative luciferase reporter activities in HUVECs transfected with control siRNA, FUS siRNA, or HDAC6 siRNA. siCtrl, control siRNA; siFUS, FUS siRNA; siHDAC6, HDAC6 siRNA. *N* = 3. ***p < 0.001 vs. siCtrl group.

As lncRNA NORAD suppressed the transcription of VEGF gene and nucleus lncRNA NORAD associated with HDAC6 *via* FUS, it was hypothesized that both HDAC6 and FUS inhibited the transcription of the VEGF gene. To prove this hypothesis, siRNA targeting HDAC6 (siHDAC6) or FUS (siFUS) was transfected into HUVECs to knock down HDAC6 or FUS expression. The efficiency of knockdown was verified by qPCR. HDAC6 expression in HUVECs transfected with siHDAC6 was knocked down by more than 70% compared with that in HUVECs treated with control siRNA (siCtrl) (Figure 4A) (*p* < 0.001).

Moreover, luciferase reporter gene assay was performed with transfection of plasmids containing VEGF gene promoter to further identify the effects of HDAC6 and FUS in VEGF expression. The luciferase activity of cells transfected with plasmids containing the *VEGF* gene promoter was increased by the transfection of siHDAC6 or siFUS compared to that of cells transfected with plasmids containing the *VEGF* gene promoter and siCtrl (Figure 4B) (*p* < 0.001). These results suggested that both HDAC6 and FUS suppressed the transcription of VEGF gene in ox-LDL-treated HUVECs.

### Nucleus LncRNA NORAD Suppresses *VEGF* Gene Transcription Through Enhancing H3K9 Deacetylation *via* Recruiting HDAC6 to *VEGF* Gene Promoter in ox-LDL-Treated HUVECs

**FIGURE 5:**
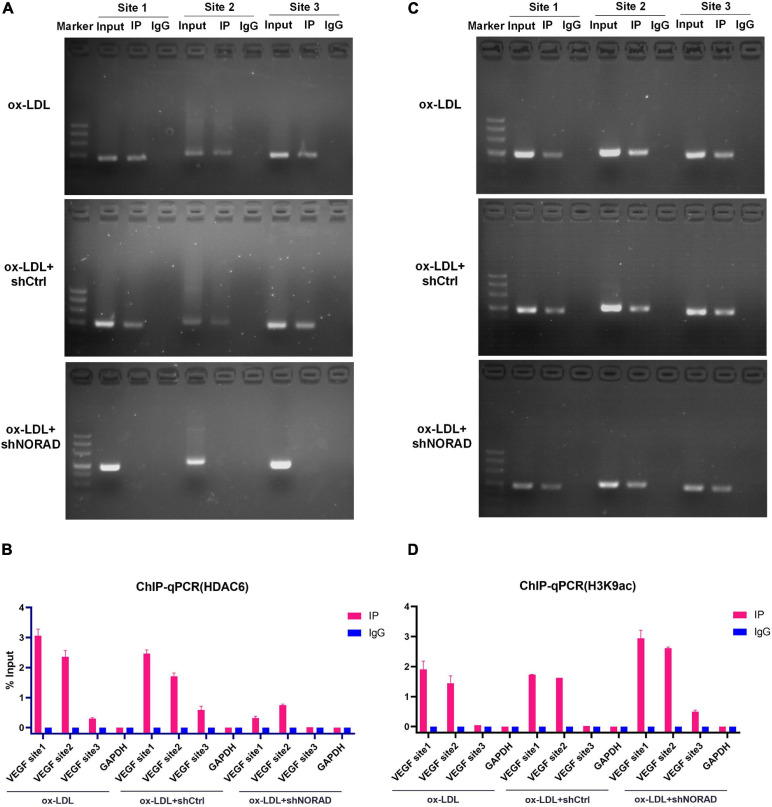
Nucleus lncRNA NORAD suppresses *VEGF* gene transcription through enhancing H3K9 deacetylation *via* recruiting HDAC6 to *VEGF* gene promoter in ox-LDL-treated HUVECs. **(A)** Immunoprecipitated chromatin was analyzed by PCR for the *VEGF* gene promoter in HUVECs transfected with control shRNA or shRNA-targeting lncRNA NORAD under ox-LDL treatment. **(B)** Quantification of HDAC6 occupancy on the *VEGF* gene promoter by qRT-PCR in HUVECs transfected with control shRNA or shRNA-targeting lncRNA NORAD under ox-LDL treatment. **(C)** Immunoprecipitated chromatin was analyzed by PCR for the *VEGF* gene promoter in HUVECs transfected with control shRNA or shRNA-targeting lncRNA NORAD under ox-LDL treatment. **(D)** Quantification of H3K9ac occupancy on the *VEGF* gene promoter by qRT-PCR in HUVECs transfected with control shRNA or shRNA-targeting lncRNA NORAD under ox-LDL treatment. shRNA; shNORAD, shRNA-targeting lncRNA NORAD. *N* = 3.

The above study had indicated that lncRNA NORAD associated with the VEGF gene promoter and nucleus lncRNA NORAD interacted with HDAC6, so we hypothesized that HDAC6 bound to the VEGF gene promoter *via* lncRNA NORAD, which served as a scaffold in ox-LDL-treated HUVECs. ChIP assay followed by PCR and qPCR was performed by HDAC6 antibody to evaluate HDAC6 binding to the VEGF gene promoter using primers spanning three sites. Results showed that most HDAC6 bound to the VEGF gene promoter at the first and second sites (Figures 5A,B). Furthermore, knockdown of lncRNA NORAD dramatically decreased the binding of HDAC6 to the VEGF gene promoter (Figures 5A,B). These results suggested that lncRNA NORAD recruited HDAC6 to the *VEGF* gene promoter.

To our knowledge, HDAC6 could deacetylate H3K9 (Ortega et al., 2021; Sun et al., 2021), and deacetylation of H3K9 at the promoter usually leads to reduced transcription (Chen et al., 2021; Kawamura et al., 2021). Therefore, ChIP assay followed by PCR and qPCR was performed by H3K9ac antibody to identify the effect of lncRNA NORAD on H3K9 deacetylation at the VEGF gene promoter in ox-LDL-treated HUVECs. Results indicated that H3K9ac was enriched in the first and second sites of the VEGF gene promoter (Figures 5C,D). Besides, knockdown of lncRNA NORAD significantly increased H3K9 acetylation at all three sites of the VEGF gene promoter (Figures 5C,D). Because HDAC6 suppressed the transcription of VEGF gene, these data suggested that nucleus lncRNA NORAD suppressed VEGF expression through enhancing H3K9 deacetylation *via* recruiting HDAC6 to *VEGF* gene promoter HDAC6 in ox-LDL-treated HUVECs.

### A Negative Feedback Regulation of FUS Expression by VEGF in HUVECs

**FIGURE 6:**
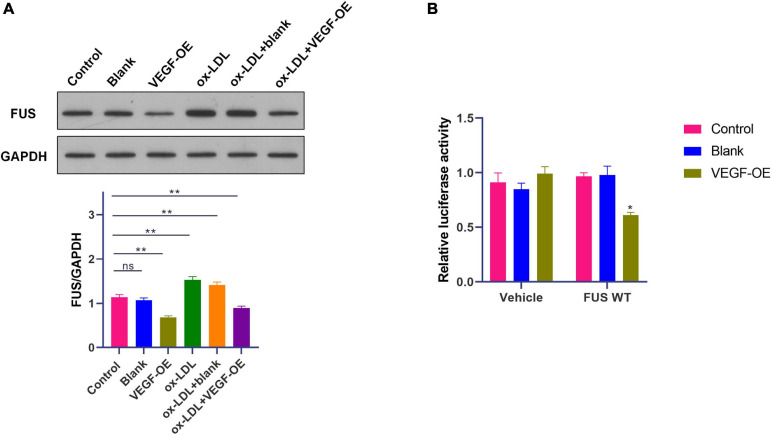
A negative feedback regulation of FUS expression by VEGF in HUVECs. **(A)** The protein level of FUS in HUVECs transfected with blank vector or VEGF expression vector under ox-LDL treatment. **(B)** Translation activity of FUS analyzed by relative luciferase reporter activities in HUVECs transfected with blank vector or VEGF expression vector. Blank, blank vector; OE-VEGF, VEGF overexpression. *N* = 3. *p < 0.05, **p < 0.01 vs. control group.

A previous study has revealed that VEGF induces degradation of cytoplasmic FUS protein in mice motor neurons (Shantanu et al., 2017). Therefore, WB was performed to identify the effect of VEGF on FUS protein expression in HUVECs. VEGF expression vector (OE-VEGF) was used to overexpress VEGF in HUVECs. Results showed that ox-LDL increased FUS protein level (*p* < 0.01), while VEGF overexpression significantly reduced FUS protein level in HUVECs treated with or without ox-LDL (Figure 6A).

Besides, luciferase reporter gene assay was performed with transfection of plasmids containing FUS ORF to further identify the effect of VEGF on FUS protein expression. The luciferase activity of cells transfected with plasmids containing FUS ORF was decreased by the transfection of OE-VEGF compared to that of cells transfected with plasmids containing FUS ORF and blank vector (Figure 6B) (*p* < 0.05). These results suggested that VEGF reduced FUS protein level by a negative feedback regulation in HUVECs.

### Knockdown of LncRNA NORAD Aggravates VEGF Expression in Thoracic Aorta of Atherosclerotic Mice

**FIGURE 7:**
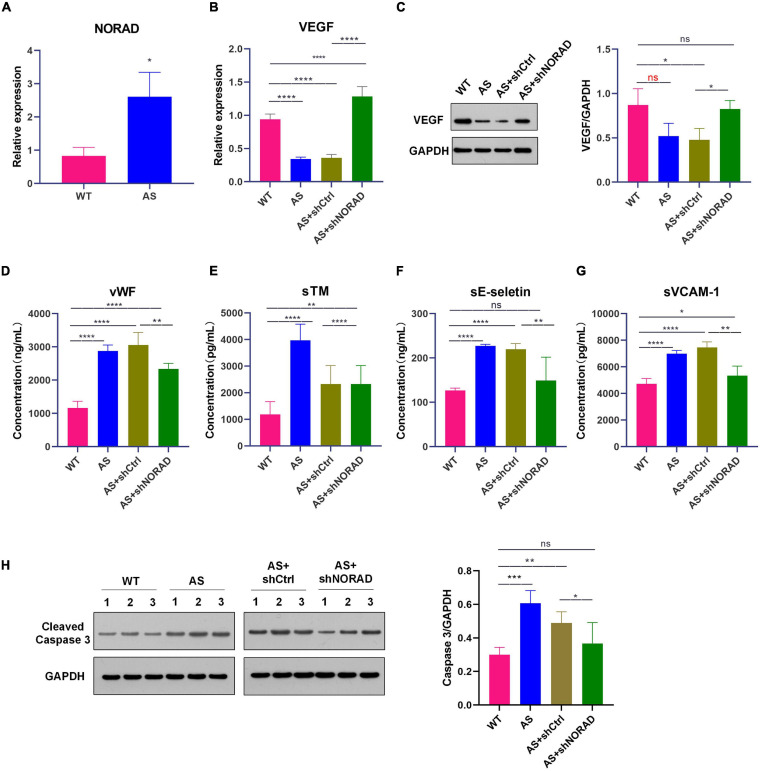
Knockdown of lncRNA NORAD aggravates VEGF expression in thoracic aorta of atherosclerotic mice. **(A)** The level of lncRNA NORAD in thoracic aorta of mice. **(B)** The mRNA level of VEGF in mice injected with control shRNA or shRNA-targeting lncRNA NORAD. **(C)** The protein level of VEGF in mice injected with control shRNA or shRNA-targeting lncRNA NORAD. **(D–G)** The level of vWF **(D)**, sTM **(E)**, sE-selectin **(F)**, and sVCAM-1 **(G)** in serum of mice injected with control shRNA or shRNA-targeting lncRNA NORAD detected by ELISA. **(H)** The level of cleaved caspase 3 in thoracic aorta of mice injected with control shRNA or shRNA-targeting lncRNA NORAD. WT, wild type; AS, atherosclerosis; shCtrl, control shRNA; shNORAD, shRNA-targeting lncRNA NORAD. *N* = 3. *p < 0.05, **p < 0.01, ***p < 0.001, ****p < 0.0001.

In this study, ApoE^–/–^ mice were used to construct an atherosclerosis animal model after 8 weeks of HFD. Results showed that lncRNA NORAD expression was upregulated in thoracic aorta of atherosclerotic (AS) mice compared to that in WT mice (Figure 7A) (*p* < 0.05). Besides, the expression of VEGF in thoracic aorta was detected by qPCR and WB. Results showed that both VEGF mRNA (*p* < 0.0001) and protein levels (*p* < 0.05) were decreased in thoracic aorta of AS mice compared to that in WT mice (Figures 7B,C), while knockdown of lncRNA NORAD induced a dramatic increase in VEGF mRNA and protein levels (Figures 7B,C). Compared to shNORAD injection, VEGF mRNA expression (*p* < 0.0001) and protein expression (*p* < 0.05) in AS mice was not affected by shCtrl injection (Figures 7B,C). These results were consistent with those of HUVECs. The above data suggested that lncRNA NORAD was upregulated to suppress VEGF expression in atherosclerosis and CAD.

### Knockdown of LncRNA NORAD Ameliorates Vascular Endothelial Injury in AS Mice

To identify whether knockdown of lncRNA NORAD attenuates vascular endothelial injury in AS mice, the serum levels of vWF, sTM, sE-selectin, and sVCAM-1 in mice were detected by ELISA. Results of ELISA showed that the serum levels of vWF (*p* < 0.0001), sTM (*p* < 0.0001), sE-selectin (*p* < 0.0001), and sVCAM-1 (*p* < 0.0001) were elevated in AS mice compared to those in WT mice, while knockdown of lncRNA NORAD dramatically reduced the serum levels of vWF, sTM, sE-selectin, and sVCAM-1 (Figures 7D–G). Compared to shNORAD injection, levels of vWF (*p* < 0.01), sTM (*p* < 0.0001), sE-selectin (*p* < 0.01), and sVCAM-1 (*p* < 0.01) in AS mice were not affected by shCtrl injection (Figures 7D–G).

Besides, cell apoptosis in thoracic aorta was identified by WB. Results showed that the level of cleaved caspase 3 (the indicator of cell apoptosis) was increased in AS mice compared to that in WT mice (*p* < 0.001), while knockdown of lncRNA NORAD dramatically reduced the level of cleaved caspase 3 (Figure 7H). Compared to shNORAD injection, cell apoptosis of thoracic aorta in AS mice was not affected by shCtrl injection (Figure 7H) (*p* < 0.05). These results suggested that knockdown of lncRNA NORAD ameliorated vascular endothelial injury in atherosclerosis *in vivo*.

### Knockdown of LncRNA NORAD Suppresses Atherosclerosis Development in AS Mice

The above data had indicated that knockdown of lncRNA NORAD ameliorated vascular endothelial injury in AS mice, and we further identified the effect of lncRNA NORAD on atherosclerosis; thus, AS mice were injected with shNORAD or shCtrl. Ultrasound imaging, Oil Red O staining, and H&E staining were utilized to evaluate the effect of lncRNA NORAD knockdown on atherosclerosis.

**FIGURE 8:**
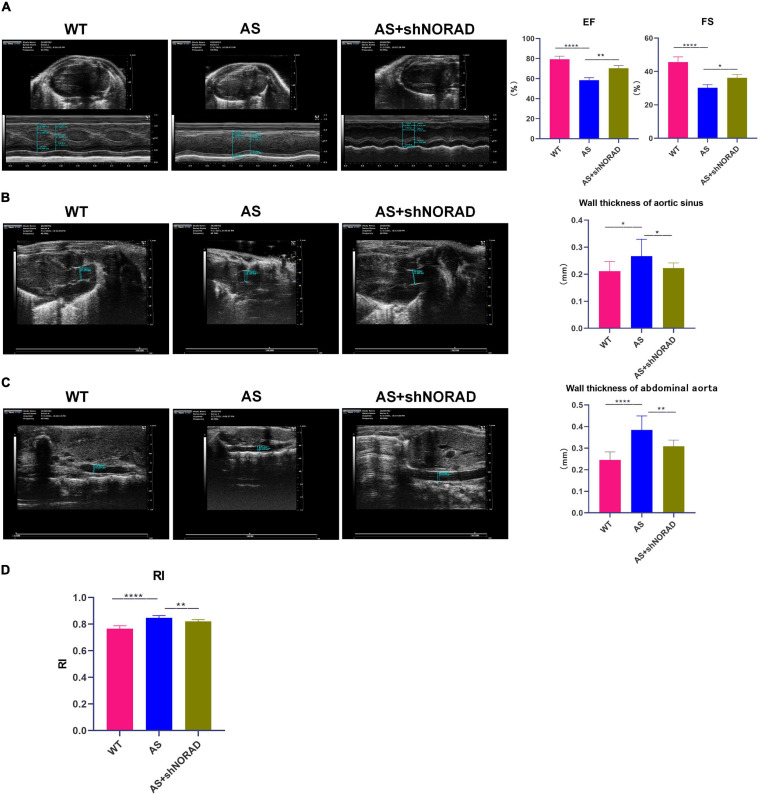
Knockdown of lncRNA NORAD improves heart function and promotes angiogenesis in atherosclerotic mice. **(A)** Representative ultrasound images of hearts in mice injected with or without shRNA-targeting lncRNA NORAD. The bar graph showed the quantification of ejection fractions (EF) or fractional shortening (FS). **(B)** Representative ultrasound images of aortic sinus in mice injected with or without shRNA-targeting lncRNA NORAD. The bar graph showed the quantification of wall thickness of aortic sinus. **(C)** Representative ultrasound images of abdominal aorta in mice injected with or without shRNA-targeting lncRNA NORAD. The bar graph showed the quantification of wall thickness of abdominal aorta. **(D)** The quantification of resistance index (RI) in mice injected with or without shRNA-targeting lncRNA NORAD. WT, wild type; AS, atherosclerosis; shCtrl, control shRNA; shNORAD, shRNA-targeting lncRNA NORAD. *N* = 3. *p < 0.05, **p < 0.01, ****p < 0.0001.

Results of ultrasound imaging showed that left ventricular ejection fractions (EF) (*p* < 0.0001) and fractional shortening (FS) (*p* < 0.0001) in AS mice were decreased compared to those in WT mice, while knockdown of lncRNA NORAD significantly increased EF and FS in AS mice (Figure 8A). By contrast, wall thickness of aortic sinus (*p* < 0.05), wall thickness of abdominal aorta (*p* < 0.0001), and resistance index (RI) (*p* < 0.0001) were increased in AS mice, and knockdown of lncRNA NORAD dramatically reduced wall thickness of aortic sinus (*p* < 0.01), wall thickness of abdominal aorta (*p* < 0.05), and RI (*p* < 0.01) in AS mice (Figures 8B–D). Compared to shNORAD injection, EF (*p* < 0.01), FS (*p* < 0.05), wall thickness of aortic sinus (*p* < 0.05), wall thickness of abdominal aorta (*p* < 0.01), and RI (*p* < 0.01) in AS mice were not affected by shCtrl injection (Figures 8A–D). These results suggested that knockdown of lncRNA NORAD improved heart function and promoted angiogenesis in AS mice.

**FIGURE 9:**
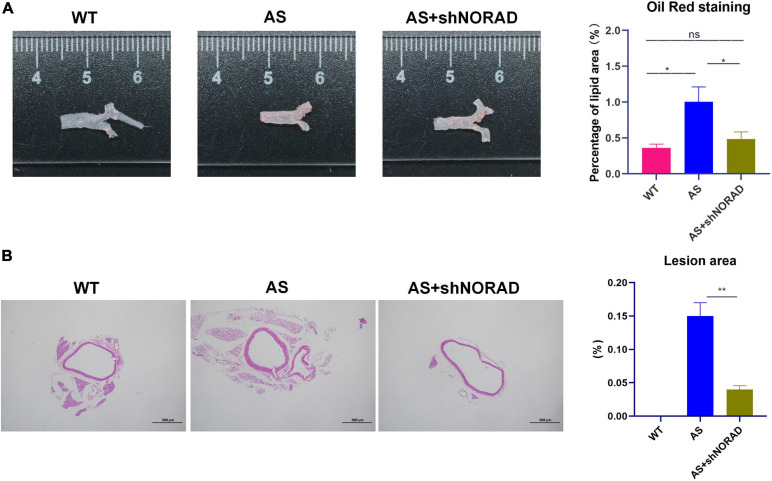
Knockdown of lncRNA NORAD suppresses atherosclerosis development in atherosclerotic mice. **(A)** Representative images of the entire aorta with Oil Red O staining. Red indicated the atherosclerotic lesion areas. The bar graph showed the quantification of percentage of lipid area. **(B)** Representative images of aorta with H&E staining. The bar graph showed the quantification of lesion area in the aorta. WT, wild type; AS, atherosclerosis; shCtrl, control shRNA; shNORAD, shRNA-targeting lncRNA NORAD. *N* = 3. Bar = 500 μm. *p < 0.05, **p < 0.01.

Moreover, results of the Oil Red O staining found that the lipid deposition area in the atherosclerotic plaque in AS mice was increased compared to that in WT mice (*p* < 0.05), whereas knockdown of lncRNA NORAD dramatically reduced the lipid deposition area (*p* < 0.05) (Figure 9A). Furthermore, H&E staining showed that the aortic plaque area was elevated in AS mice compared to that in WT mice, while knockdown of lncRNA NORAD decreased the aortic plaque area (*p* < 0.01) (Figure 9B). Compared to shNORAD injection, lipid accumulation (*p* < 0.05) and aortic plaque (*p* < 0.01) in AS mice were not affected by shCtrl injection (Figures 9A,B). Therefore, all these data suggested that knockdown of lncRNA NORAD attenuated atherosclerosis in AS mice.

## Discussion

This study indicated the effect of lncRNA NORAD on vascular endothelial cell injury and atherosclerosis and potential mechanisms. First, lncRNA NORAD expression was increased in ox-LDL-treated HUVECs and thoracic aorta of AS mice, and knockdown of lncRNA NORAD alleviated vascular endothelial cell injury and atherosclerosis development *in vitro* and *in vivo*. Second, knockdown of lncRNA NORAD aggravated ox-LDL-reduced or atherosclerosis-decreased VEGF expression in HUVECs and thoracic aorta of mice to ameliorate vascular endothelial cell injury and atherosclerosis development. Moreover, nucleus lncRNA NORAD suppressed *VEGF* transcription through enhancing H3K9 deacetylation *via* recruiting HDAC6 to the *VEGF* gene promoter in ox-LDL-treated HUVECs. In addition, VEGF reduced FUS expression by a negative feedback regulation in HUVECs.

LncRNA NORAD is a novel lncRNA, and the role of lncRNA NORAD in vascular endothelial cell injury, atherosclerosis, and CAD remains unclear. This study demonstrated that lncRNA NORAD induced vascular endothelial cell injury and atherosclerosis through suppressing *VEGF* transcription by enhancing H3K9 deacetylation. Numerous studies have revealed that lncRNA NORAD usually regulates the expression of target genes as a competing endogenous RNA (ceRNA). For instance, lncRNA NORAD promotes pancreatic cancer metastasis through enhancing epithelial–mesenchymal transition *via* targeting hsa-miR-125a-3p to increase RhoA expression (Li et al., 2017). Moreover, lncRNA NORAD increases the sensitivity of osteosarcoma to cisplatin through targeting miR-410-3p (Xie et al., 2020). In addition, lncRNA NORAD upregulates TLR4 expression *via* targeting miR-520h to promote the progression of diabetic nephropathy (Qi et al., 2020). Besides, lncRNA NORAD also exerts decoy function by sequestering target proteins to regulate genomic stability or cancer metastasis (Lee et al., 2016; Tan et al., 2019). However, no studies have reported the effect of lncRNA NORAD on regulating the transcription of target gene *via* modifying histone modification. Therefore, this study revealed the effect of lncRNA NORAD on regulating the transcription of target gene through recruiting histone modification enzyme to the promotor of target gene for the first time.

Though the suppressive role of lncRNA NORAD in VEGF expression was uncovered for the first time in the present study, the effects of lncRNAs on VEGF expression have been demonstrated. For example, LncRNA TDRG1 directly associates with VEGF protein to increase VEGF protein and promotes the progression of endometrial carcinoma (Chen et al., 2018). Furthermore, lncRNA ROR promotes tumorigenicity in renal cell carcinoma through upregulating VEGF expression *via* targeting miR-206 as a ceRNA (Shi et al., 2019). Moreover, lncRNA SNHG12 increases VEGF expression by targeting miR-150 to induce angiogenesis after ischemic stroke (Zhao et al., 2018). However, the effects of lncRNAs on regulating *VEGF* gene transcription have not been reported. Thus, our study indicated the effects of lncRNAs on regulating *VEGF* gene transcription *via* modifying histone modification for the first time.

To date, the role of HDAC6 in VEGF expression remains unclear. Previous studies have revealed the effects of HDACs but not HDAC6 on *VEGF* gene transcription. As a transcriptional inhibitor of *VEGF* gene, Kruppel-like Factor 4 (KLF4) recruits HDAC2 and HDAC3 to the *VEGF* gene promoter for suppressing *VEGF* gene transcription in breast cancer (Ray et al., 2013). Moreover, HDAC3 plays a negative role in angiogenesis *via* inhibiting VEGF expression (Park et al., 2014). In addition, HDAC4 decreases VEGF expression to regulate neuronal morphology (Litke et al., 2018). Therefore, the finding of this study that HDAC6 suppressed VEGF expression is reliable.

LncRNA NORAD and HDAC6 are located in both the cytoplasm and nucleus. Cytoplasmic lncRNA NORAD interacts with PUMILIO protein to regulate cell mitosis through regulating levels of PUMILIO-targeted mRNAs (Tichon et al., 2016). In addition, HDAC6 regulates the acetylation of numerous non-histone proteins in the cytoplasm (LoPresti, 2020). For instance, HDAC6-mediated deacetylation of Miro1 suppresses axon growth (Kalinski et al., 2019). Except for nucleus lncRNA NORAD and HDAC6, cytoplasmic lncRNA NORAD and HDAC6 may also play a suppressive role in VEGF expression through associating with VEGF protein and subsequently enhancing the deacetylation of VEGF protein in vascular endothelial cell injury, atherosclerosis, and CAD.

Our study also identified a negative feedback loop between VEGF protein and FUS protein as VEGF protein could decrease FUS protein level. Therefore, we guessed when the VEGF expression was decreased, the remaining VEGF protein would interact with FUS protein followed by the degradation of FUS protein to inhibit the recruitment of HDAC6 to the *VEGF* gene promoter by lncRNA NORAD, and finally, VEGF expression would be elevated to attenuate vascular endothelial cell injury.

However, the present study still had some limitations. First, the size of groups in animal studies was small, and more mice would be utilized in future studies. Second, the potential mechanisms on how lncRNA NORAD regulates vascular endothelial cell injury and atherosclerosis should be further confirmed by an *in vivo* study.

In summary, this study indicated that knockdown of ox-LDL-induced or atherosclerosis-increased lncRNA NORAD aggravated VEGF expression to ameliorate vascular endothelial cell injury and atherosclerosis development, and nucleus lncRNA NORAD suppressed *VEGF* transcription through enhancing H3K9 deacetylation *via* recruiting HDAC6 to the *VEGF* gene promoter in ox-LDL-treated HUVECs. In addition, VEGF reduced FUS expression by a negative feedback regulation in HUVECs. The findings of our study could facilitate discovering novel diagnostic markers and therapeutic targets for CAD.

## Data Availability Statement

The original contributions presented in the study are included in the article/Supplementary Material, further inquiries can be directed to the corresponding author/s.

## Ethics Statement

The studies involving human participants were reviewed and approved by the Changzhou Second People’s Hospital Affiliated to Nanjing Medical University. Written informed consent for participation was not required for this study in accordance with the national legislation and the institutional requirements. The animal study was reviewed and approved by the Changzhou Second People’s Hospital Affiliated to Nanjing Medical University. Written informed consent was obtained from the individual(s) for the publication of any potentially identifiable images or data included in this article.

## Author Contributions

CP, HK, and QW contributed to the conception and design of the study and wrote sections of the manuscript. RY, XT, and HS organized the database. TW and MZ performed the statistical analysis. CP, HK, and QW wrote the first draft of the manuscript. All authors contributed to manuscript revision, read, and approved the submitted version.

## Conflict of Interest

The authors declare that the research was conducted in the absence of any commercial or financial relationships that could be construed as a potential conflict of interest.

## Footnotes

1 http://starbase.sysu.edu.cn/

## Supplementary Material

Supplementary Figure 1 Representative images of silver staining for RAP assays. **(A–C)** Representative images of silver staining for RAP assays performed in ox-LDL-treated HUVECs **(A)**, HUVECs transfected with control shRNA **(B)**, or HUVECs transfected with shRNA-targeting lncRNA NORAD **(C)**. NC, negative control.

Supplementary Figure 2 Representative images of silver staining for Co-IP performed in nucleus of ox-LDL-treated HUVECs. Ab, antibody.

Supplementary Figure 3 The mRNA level of FUS in HUVECs transfected with control siRNA or FUS siRNA. siCtrl, control siRNA; siFUS, FUS siRNA. *N* = 3. ^****^*P* < 0.0001 vs. siCtrl group.
