# Supplementary figures and images for: LncRNA NORAD Promotes Vascular Endothelial Cell Injury and Atherosclerosis Through Suppressing VEGF Gene Transcription via Enhancing H3K9 Deacetylation by Recruiting HDAC6
Source: Front Cell Dev Biol. 2021 Jul 9;9:701628. doi: 10.3389/fcell.2021.701628 (PMC8301222; doi:10.3389/fcell.2021.701628)

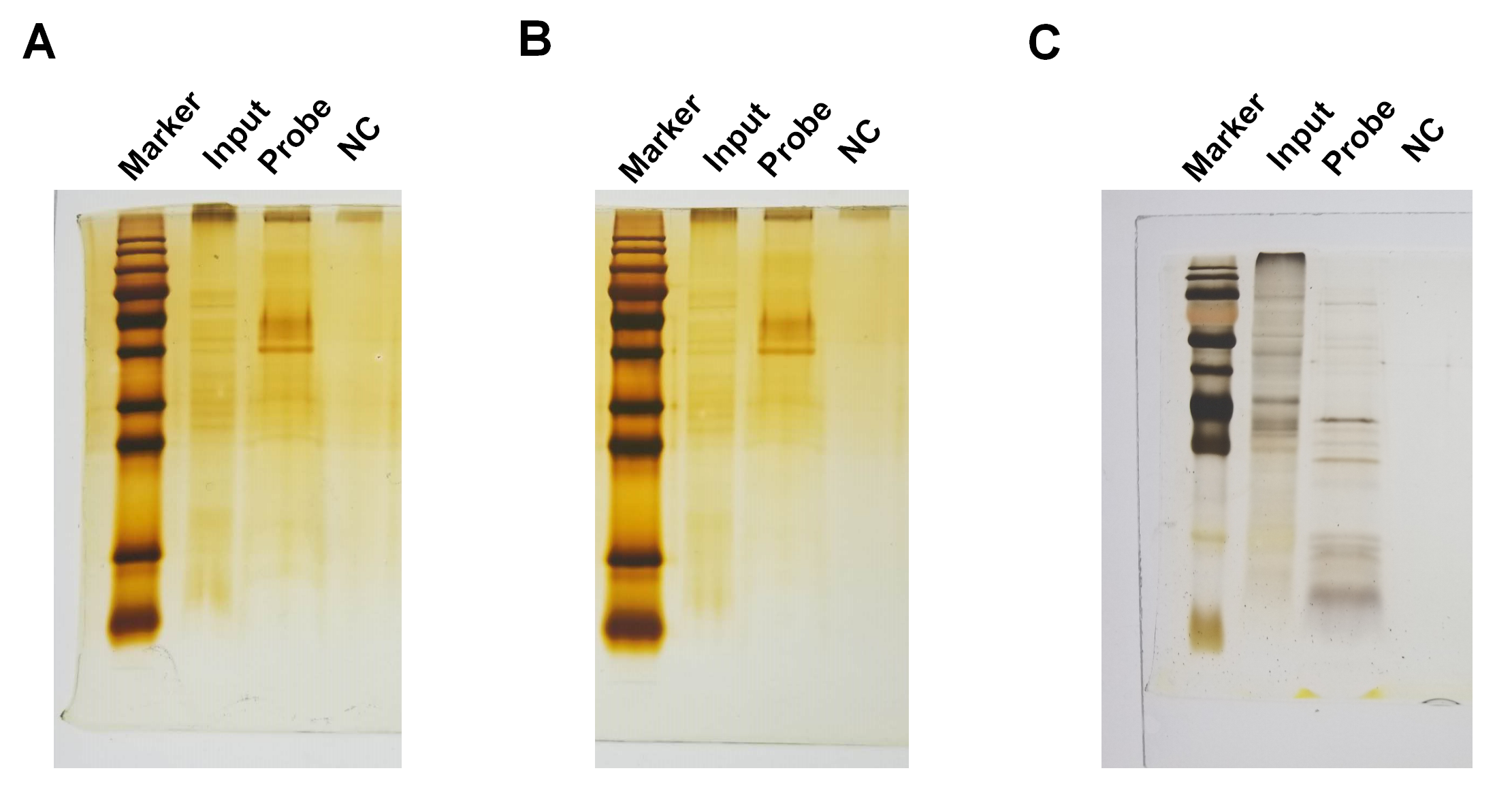

Supplement: Supplementary Figure 1 — Representative images of silver staining for RAP assays. (A–C) Representative images of silver staining for RAP assays performed in ox-LDL-treated HUVECs (A), HUVECs transfected with control shRNA (B), or HUVECs transfected with shRNA-targeting lncRNA NORAD (C). NC, negative control. [file Image_1.TIF]

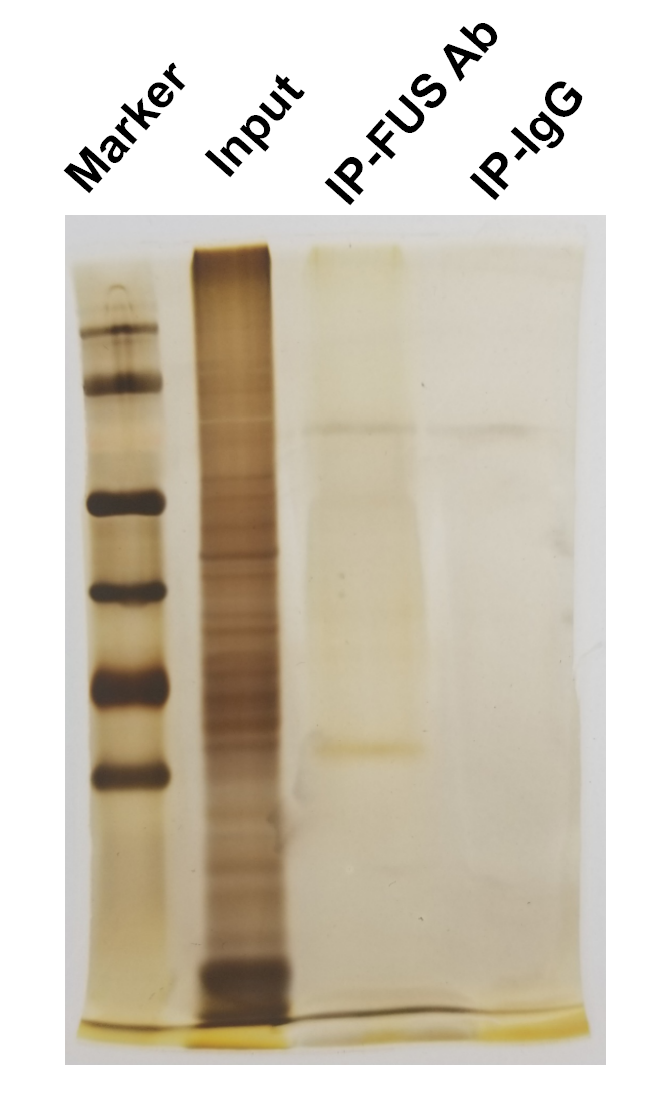

Supplement: Supplementary Figure 2 — Representative images of silver staining for Co-IP performed in nucleus of ox-LDL-treated HUVECs. Ab, antibody. [file Image_2.TIF]

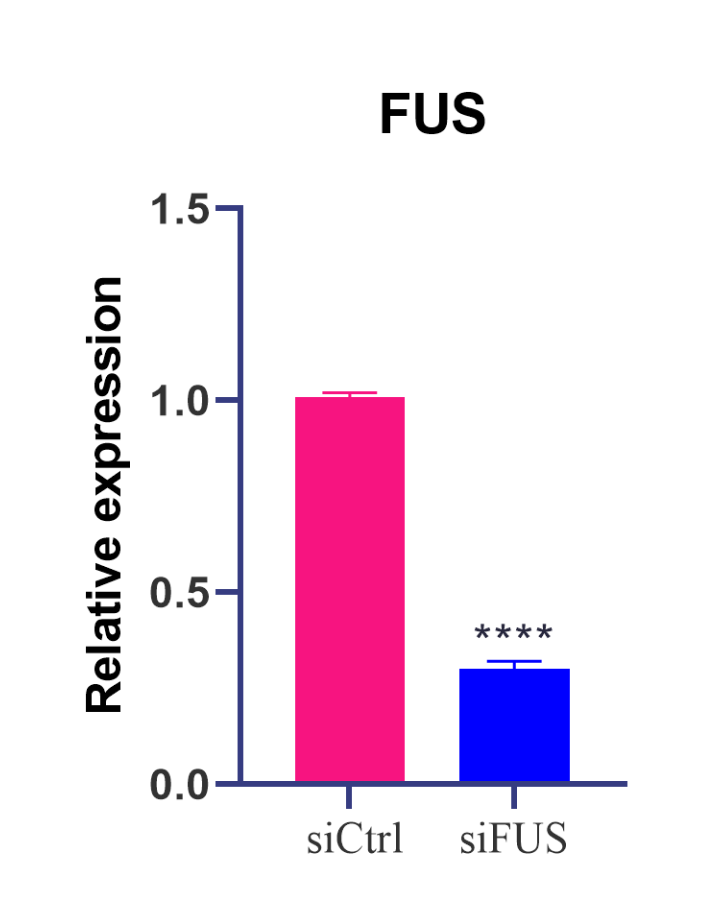

Supplement: Supplementary Figure 3 — The mRNA level of FUS in HUVECs transfected with control siRNA or FUS siRNA. siCtrl, control siRNA; siFUS, FUS siRNA. N = 3. ****P < 0.0001 vs. siCtrl group. [file Image_3.TIF]
